# Self-Photoluminescence of Unzipped Multi-Walled Carbon Nanotubes

**DOI:** 10.3390/nano11071632

**Published:** 2021-06-22

**Authors:** Mengyao Chen, Xiaohua Qi, Wenna Zhang, Na Yang, Di Yang, Yao Wang, Lixiu Zhang, Wenbin Yang, Linjun Huang, Miaorong Zhang, Shichao Wang, Peter Strizhak, Jianguo Tang

**Affiliations:** 1 National Center of International Joint Research for Hybrid Materials Technology, Institute of Hybrid Materials, National Base of International Sci. & Tech. Cooperation on Hybrid Materials, College of Materials Science and Engineering, Qingdao University, 308 Ningxia Road, Qingdao 266071, China; chenmengyao210@163.com (M.C.); qixiaohua0930@163.com (X.Q.); zhangwenna1345@163.com (W.Z.); 17669490805@163.com (N.Y.); yd130102@163.com (D.Y.); wangyaoqdu@126.com (Y.W.); zhang15506460358@163.com (L.Z.); 2018025335@qdu.edu.cn (W.Y.); huanglinjun@qdu.edu.cn (L.H.); mrzhang2018@qdu.edu.cn (M.Z.); wangsc@qdu.edu.cn (S.W.); 2 L.V. Pysarzhevsky Institute of Physical Chemistry, National Academy of Sciences of Ukraine, 31 Prosp. Nauky, 03028 Kyiv, Ukraine

**Keywords:** unzipped carbon nanotubes, oxygen-containing functional group, band gap, self-photoluminescence

## Abstract

Unzipping of carbon nanotubes (CNTs) has been widely explored to obtain new nanocarbon structures with promising properties. In this work, we report that unzipping of CNTs according to the well-established modified Hummers method produces unzipped CNTs (uCNTs) that exhibit self-photoluminescence that depends on the diameter of pristine CNTs. The uCNTs were characterized using FTIR spectroscopy, XRD, XPS, and Raman spectroscopy indicating that unzipping is accompanied by the introduction of defects and oxygen-containing functional groups. The morphology of CNTs and uCNTs was determined by TEM showing longitude unzipping of CNTs. Our study shows that increasing the diameter of pristine CNTs results in decreasing the edge etching effect and decreasing the functionality of uCNTs. Based on the UV-Vis spectra, the band gap of uCNTs was calculated using the Kubelka–Munk function. The band gap of uCNTs increased with decreasing diameter of pristine CNTs. The uCNTs exhibited photoluminescence with a good emission in the visible light region. The uCNTs with the largest band gap and the highest oxygen content had the strongest fluorescence intensity. Moreover, different metal ions produced different degrees of fluorescence quenching for uCNT-15, which verified the self-photoluminescence of uCNTs.

## 1. Introduction

Carbon nanotubes (CNTs) have attracted much attention based on their unique structure and outstanding performance [1]. Contrary to most conventional materials, CNTs have salient optical features mainly including various energy bands and specific laws of carrier migration, transition, and recombination. Recently, researchers have focused on the fluorescence phenomenon of carbon nanotubes [2]. The high sensitivity of CNTs to light is attributed to their unique chemical structure, which can absorb light of all wavelengths. Furthermore, different diameters and aspect ratios of CNTs result in different conjugated π electron cloud densities, causing varying degrees of light absorption and multiple optical properties [3]. CNTs are single-wall (SWCNT) or multi-wall (MWCNT). SWCNTs can emit bright fluorescence in the near-infrared wavelength range from 800 to 1600 nm in an aqueous micelle suspension [4,5]. MWCNTs do not show fluorescence under ultraviolet light without proper treatment. However, MWCNTs can produce luminescence following one of two main strategies. Mixing MWCNTs with a polymer in an organic or aqueous solution and adding a surfactant to make the carbon nanotubes covalently connect with the soluble linear polymer results in the MWCNTs bonding to a polymer show luminescence [6]. The binding effect of surfactants in an organic solvent is more negative than in aqueous solutions, and the surfactant is affected by impurities easily [7]. Another strategy is based on a modification or functionalization of the MWCNTs’ surface followed by grafting some luminescent ions or groups to make CNTs emit light [8,9]. However, this way may deteriorate the performance of the CNTs [10]. It has been recently reported that the functional groups on the surface of unzipped carbon nanotubes (uCNTs) are utilized to anchor rare earth complexes through coordination reaction, generating a fluorescent hybrid material with pronounced fluorescence characteristics in the ultraviolet and visible light regions [11,12]. At present, there still exist immense challenges for exploring the self-photoluminescence properties of multi-walled CNTs in the visible light range.

The structure of MWCNTs significantly differs from the structure of SWCNTs due to the coupling effect between layers of MWCNTs, which facilitates the formation of a smaller band gap [13]. Subsequently, most of the excited electrons transform energy to non-radiative transitions under the rapid vibration of adjacent tubes, making it difficult to observe fluorescence. In this work, we found that the unzipping of MWCNTs gives a good strategy to produce MWCNT derivatives that exhibit photoluminescence. Following this strategy, MWCNTs were unzipped and a large number of oxygen-containing functional groups were introduced to the uCNTs’ surface by the modified Hummers method. The energy level of uCNTs can be improved and band gap can be enhanced by the oxygen-containing functional groups. Furthermore, the twisted carbon atoms are attached to oxygen-containing functional groups, producing many disorder-induced local states and inducing the optical transitions [14]. To explore the fluorescence principle of multi-walled carbon nanotubes, we studied multi-walled CNTs with inconsistent outer diameters and unzipped them under the same conditions. TEM, FTIR, XRD, XPS, UV-Vis, and Raman spectroscopy were adopted to thoroughly explore the structure of uCNTs. This allowed us for the first time to discuss the optical properties of uCNTs in detail including band gap and photoluminescence. We also show that the metal ions can be used for the production of fluorescence quenching properties of uCNTs, verifying the self-photoluminescence of uCNT.

## 2. Materials and Methods

### 2.1. Materials

KMnO_4_, H_2_SO_4_, HCl, and H_2_O_2_ were supplied from Shanghai Macleans Biochemical Technology Co., Ltd. (Shanghai, China) and did not require further processing. Three different types of multi-wall carbon nanotubes were purchased from Xianfeng Nano Material Technology Co., Ltd. (Jiangsu, China): CNT-15 (purity > 95%; outside diameter: 10–20 nm; length: 0.5–2 μm), CNT-23 (purity > 95%; outside diameter: 20–30 nm; length: 0.5–2 μm), and CNT-45 (purity > 95%; outside diameter: 40–50 nm; length: 0.5–2 μm).

### 2.2. Experimental Section

**Figure 1:**
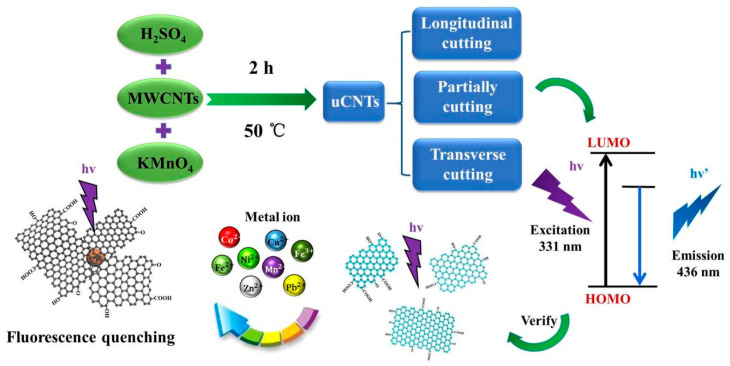
Illustration of the formation process of uCNT and its corresponding energy.

CNTs were unzipped according to the procedure described elsewhere [15,16,17]. As shown in Figure 1, the schematic diagram reveals the typical uCNT preparation processes in which the multi-walled carbon nanotubes can be converted into uCNTs by a traditional Hummers method [18]. However, in the actual conditions, there are undesired unzipping process such as ‘cuttings’ behavior, which would induce the mixing of uCNTs with layer ‘cuttings’. First, 200 mg of original CNTs were added into 35 mL of concentrated sulfuric acid, the mixture was stirred for 10 min using a magnetic stirrer, then ultrasonically dispersed for 8 h at room temperature until a homogeneous solution was acquired. Then, 1.0 g KMnO_4_ with the same amounts was added into the solution and stirred, and added 5 times respectively, 10 min apart, and further stirred for 2 h at 50 °C. The reaction mixture was added very slowly to ice containing 35 mL of H_2_O_2_ (30%) under ice bath conditions. Subsequently, an appropriate amount of H_2_O_2_ (30%) was added dropwise into the reaction mixture until the gas was not observed. Then, the mixture was washed and centrifuged with HCl (the ratio of distilled water to hydrochloric acid was 3:1) and distilled water until pH was 4. Finally, the black powder was collected and dried in an oven at 60 °C. The obtained unzipped CNTs are labeled as uCNTs accordingly for each sample: uCNT-15, uCNT-23, and uCNT-45.

### 2.3. Characterization

The solution and the solid obtained after the synthesis were characterized by using various techniques, including transmission electron microscopy (TEM), X-ray diffraction (XRD), X-ray photoelectron spectra (XPS), FTIR spectra, Raman spectra, UV–Vis spectroscopy, and photoluminescence (PL) spectra. TEM images were used to analyze the microstructure of the original CNTs and unzipped CNTs by JEOL 2011 (JEOL Ltd., Tokyo, Japan). The XRD study was performed in a wide-angle range (2θ = 10–80°) by Bruker D8 (Bruker, Beijing, China) Advance model at a scanning rate of 2°/min, with monochromatized Cu, Kα radiation (k = 1.5406 Å). XPS confirmed the chemical compositions of samples with an ESCALAB 250 spectrometer (Thermo Fisher Scientific, Waltham, MA, USA). The FTIR spectra were recorded on a MAGNA-IR 550 (Nicolet Instrument Corporation, Madison, WI, USA) and the spectrum was obtained by mixing the sample with KBr. Raman spectroscopy was performed with an Almega Thermo Nicolet Dispersive Raman Spectrometer (Thermo Fisher Scientific, Waltham, MA, USA) with a 532 nm laser excitation and 2.0 mW laser energy. The optical properties were recorded in the wavelength range from 200 to 800 nm by UV–Vis spectroscopy (Lambda 750S, PerkinElmer, Waltham, MA, USA). PL spectroscopy was performed with an FLS1000 (Edinburgh Instruments Ltd., Edinburgh, United Kingdom).

## 3. Results and Discussion

**Figure 2:**
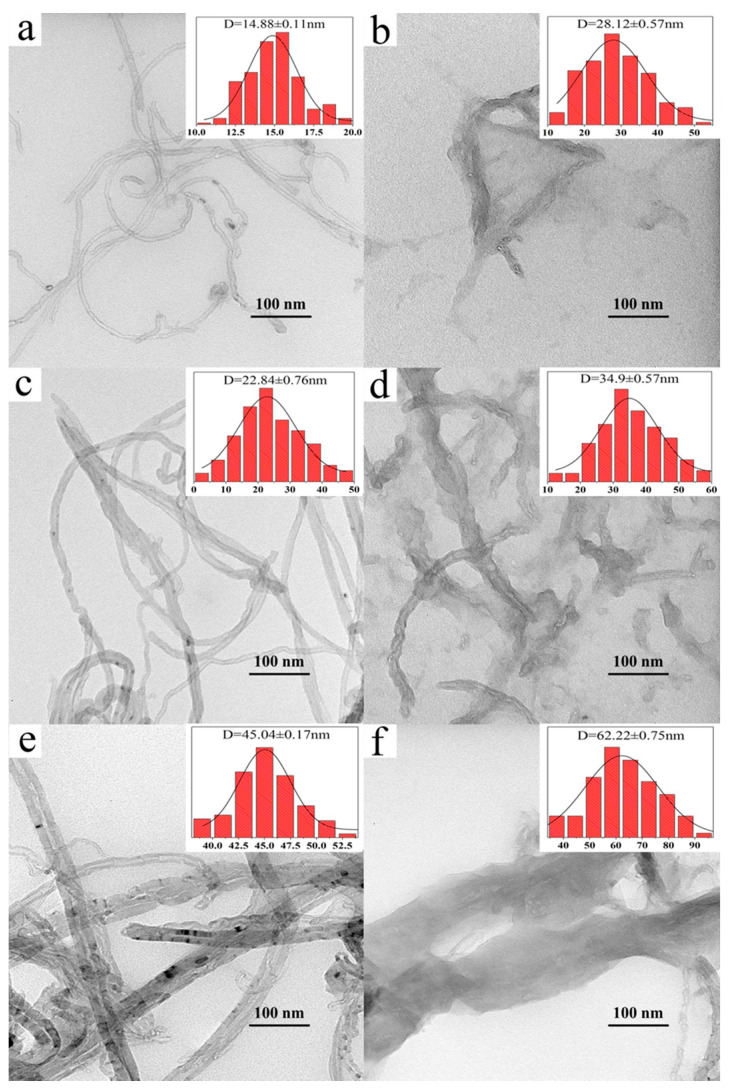
TEM images and the distributions of CNT-15 (**a**), uCNT-15 (**b**), CNT-23 (**c**), uCNT-23 (**d**), CNT-45 (**e**), and uCNT-45 (**f**).

Figure 2a,c,e gives the TEM images of CNT-15, CNT-23, and CNT-45, respectively. Figure 2b,d,f gives the images of corresponding uCNTs, which are uCNT-15, uCNT-23, and uCNT-45, respectively. Table 1 presents the values of average diameters CNTs, the average width of uCNTs, values of corresponding FWHM, and the etching level. The MWCNTs samples are labeled as CNT-R where R is an outer diameter of MWCNTs. The corresponding uCNTs are labeled as uCNT-R. Figure 2a,b indicates that CNT-15 had a hollow tubular structure, the two ports were closed, the surface was relatively smooth, and the outer diameter was 14.88 ± 0.11 nm, which shows a strong tendency to agglomerate. Unzipping of CNT-15 expanded the surface into graphene nanoribbons with a width of 28.12 ± 0.57 nm, which was almost twice that of CNT-15. This shows that the CNT-15 was successfully unzipped, causing the tubular structure of the CNT-15 to break and the surface area to increase [19]. Figure 2c–f shows that the outer diameters of CNT-23 and CNT-45 were 22.84 ± 0.76 nm and 45.04 ± 0.17 nm, respectively. The widths of uCNT-23 and uCNT-45 were 34.9 ± 0.57 nm and 62.22 ± 0.75 nm, respectively. Compared to uCNT-15, the width of uCNT was not nearly twice the outer diameter of the untreated CNTs. If the CNT of an outer diameter D was purely unzipped longitudinally, a width of uCNT would be expected to be W = πD. A value of E = (1 − W/πD) * 100% may be referred to as a level of the edge etching of uCNTs during the unzipping process. Table 1 shows that increasing the diameter of CNTs increased the value of E, i.e., etching of edges increased with increasing the CNT diameter. The surface of pristine CNTs is smooth, and the edges of uCNTs are rough. In the process of unzipping, the surface of the CNTs was etched by the oxidant of KMnO_4_, which caused severe harm and distortion of the carbon network forming uCNTs of different cutting degrees, abundant defects, and oxygen-containing groups [20].

**Figure 3:**
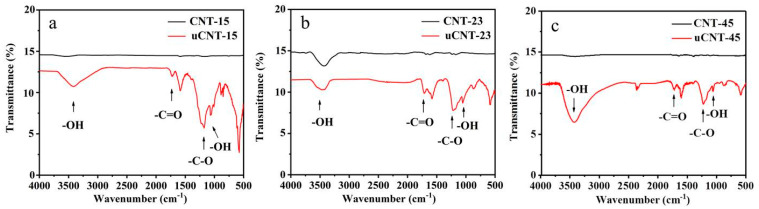
FTIR spectra of CNT-15 and uCNT-15 (**a**), CNT-23 and uCNT-23 (**b**), CNT-45 and uCNT-45 (**c**).

The uCNTs had high dispersion stability, which may have been caused by the introduction of the oxygen-containing functional groups during unzipping. The characteristic functional groups in the samples were analyzed by infrared spectroscopy. Figure 3 shows the FTIR spectra of CNTs and corresponding uCNTs. Pristine CNTs showed some weak peaks. uCNTs showed a -OH stretching vibration peak around 3400 cm^−1^ mainly caused by the adsorbed water. The absorption peak of the -OH functional group introduced by the acid treatment appeared at about 1050 cm^−1^, a peak of -C-O stretching vibration appeared at 1220 cm^−1^. A stretching vibration peak at about 1720 cm^−1^ corresponds to the absorption of -C=O bond in carboxyl group [21,22]. Therefore, the FTIR study showed that unzipping introduced a large number of oxygen-containing functional groups on the surface of uCNTs. It is worth noting that the intensity of tensile vibration peaks representing -C=O, -C-O, and -OH groups depended on the diameter of pristine CNTs. Therefore, as it follows from the previous section, it depended on the level of etching. The intensity of these vibration peaks was the highest for uCNT-15 and the lowest for uCNT-45. As a result, the smaller the outer diameter of CNTs and the lower the level of the edge etching, the more oxygen-containing functional groups were introduced.

**Figure 4:**
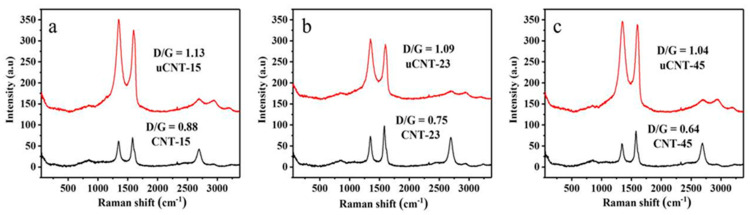
Raman spectra of CNT-15 and uCNT-15 (**a**), CNT-23 and uCNT-23 (**b**), CNT-45 and uCNT-45 (**c**).

The Raman spectra are shown in Figure 4 highlighting the structural changes for CNTs and uCNTs. There were four peaks in the CNTs and uCNTs spectra, which were the D peak around 1349 cm^−1^, the G peak around 1586 cm^−1^, the 2D peak around 2696 cm^−1^, and the 2G peak around 2943 cm^−1^. The first-order Raman scattering peaks were D peak and G peak, and the second-order Raman peaks were 2D peak and 2G peak [23]. After unzipping, the 2D band of the CNTs was significantly weakened in the spectra of uCNTs. This indicates that the structure of uCNTs was more disordered and the number of defects increased during unzipping [24]. The D peak was caused by sp^3^ hybridization of CNTs or uCNTs at the defect site, and the G peak was caused by sp^2^ hybridization of regularly ordered carbon atoms. Accordingly, the ratio of the intensity of D peak to G peak (R = I_D_/I_G_) gives the degree of structural regularity. The 2D peak was caused by double resonance Raman scattering and had a strong signal in the complete structure. The R values for CNT-15, CNT-23, and CNT-45 were 0.88, 0.75, and 0.64, respectively. After the CNTs were unzipped, their R values rose to 1.13, 1.09, and 1.04, respectively. This indicates that some sp^3^ carbon was formed by oxidation, and the larger the R-value, the higher the sp^3^ carbon content formed [15]. The Raman spectrum clearly shows the typical sp^3^ and sp^2^ hybridization corresponding to the defect sites and carbon atoms, respectively. Meanwhile, the infrared spectrum presents the composition information of C-O, C=O, -OH, and other functional groups. The resulting consequence demonstrates that the Raman spectroscopy and infrared spectroscopy complement each other.

**Figure 5:**
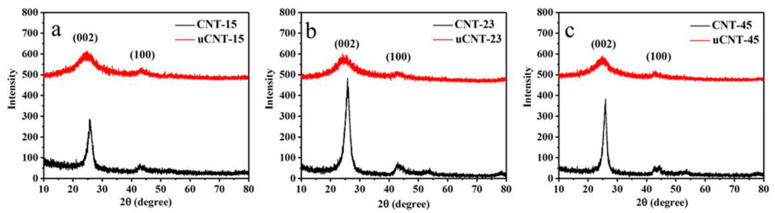
X-ray diffraction patterns of CNT-15 and uCNT-15 (**a**), CNT-23 and uCNT-23 (**b**), CNT-45 and uCNT-45 (**c**).

Figure 5 shows the XRD spectra of CNTs and uCNTs. Table 2 describes the FWHM of all samples around 26° and 43°. Figure 5 shows that two peaks in the XRD spectra of CNTs appeared around 26° and 43°. The peak at 26° corresponds to the graphite-like phase (002) diffraction peak (d_002_ = 3.4 Å) and the peak at 43° represents the characteristic diffraction peak of the (100) plane. The XRD spectra of uCNTs were characterized by two characteristic peaks at 26° and 43°. Compared to the characteristic peaks of the CNTs spectra, the peaks of uCNTs were not so sharp. After unzipping, the 002 peak was broadened and shifted to a lower 2θ value for all samples. This indicates that the carbon structure of CNTs can be disturbed by oxygen-containing functional groups [25]. Table 2 gives the half-widths of the diffraction peaks for each sample. The half-width of the diffraction peak of uCNTs around 26° was significantly higher compared to CNTs. This may be related to the destruction of the ordered and multi-walled structure of CNTs. The effect was more pronounced for uCNT-15 and less pronounced for uCNT-45. This shows that the oxidation method had a damaging effect on the surface of CNTs, that is, the CNTs reacted more violently during the redox process that involved potassium permanganate. Therefore, the structure of the surface was damaged, the integrity of its lattice structure was destroyed, and the half-width of the diffraction peak was widened. The smaller the outer diameter of the CNT, the greater the degree of damage to its structure observed.

**Figure 6:**
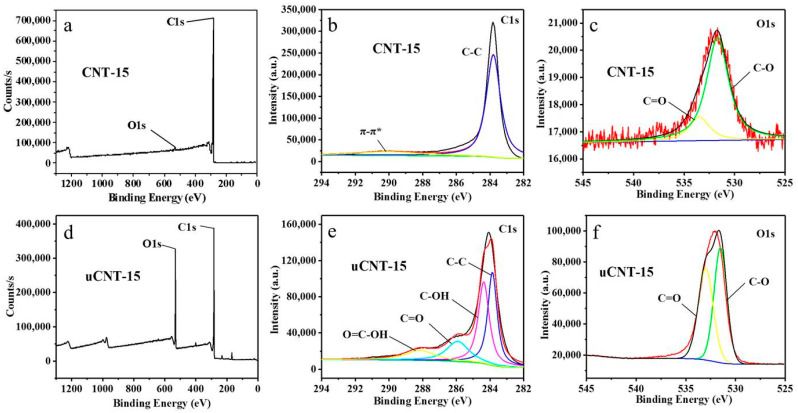

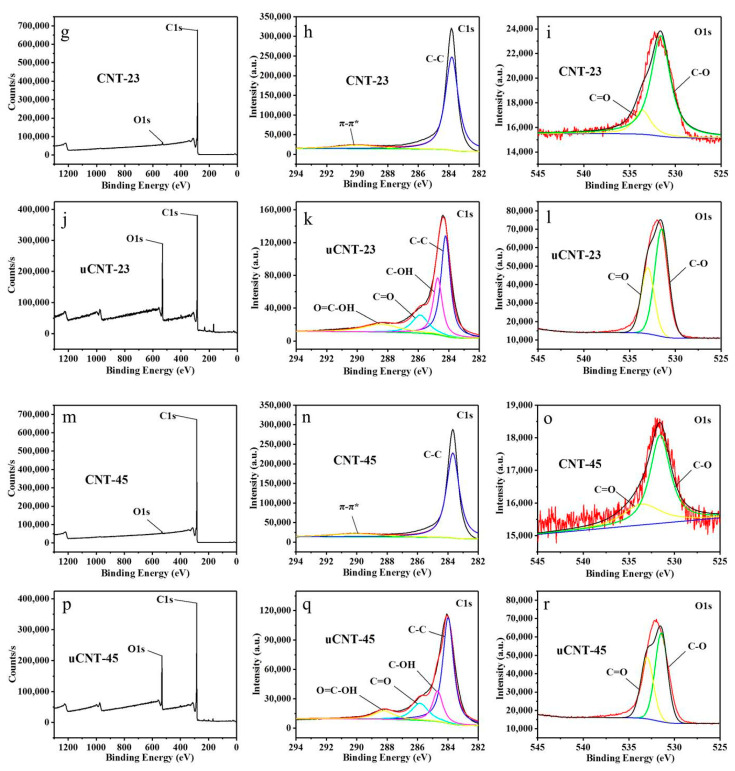
XPS spectra of CNT-15 (**a**–**c**), uCNT-15 (**d**–**f**), CNT-23 (**g**–**i**), uCNT-23 (**j**–**l**), CNT-45 (**m**–**o**), and uCNT-45 (**p**–**r**).

An XPS study was performed to further compare element composition and content distribution information. The total spectra and percentage of element content of CNTs and uCNTs are shown in Figure 6 and presented in Table 3. In addition to O1s and C1s signals, the spectra of uCNTs also contained the weak peaks S2p at 170 eV and S2s at 233 eV indicating a presence of sulfur impurities. The oxygen content and O/C ratio significantly increased after unzipping. The oxygen content and O/C ratio of uCNTs increased with a decrease in the diameter of CNT. As shown in the O1s spectrum, CNTs and uCNTs had two peaks at 531.5 eV and 533 eV, respectively. The binding energy signal at 531.5 eV is assigned to the C-O in the surface lattice oxygen, while the peak at 533 eV is attributed to the oxygen-containing group C=O in the sample. Moreover, the peaks of the two peaks of uCNTs were much larger than the peaks of the two peaks of CNTs. This may be related to a smaller specific surface area of CNTs with a larger outer diameter, which led to a lower oxidation degree. The C1s spectrum of CNT presented in Figure 6b was deconvoluted into two feature peaks of CNTs, a strong peak at 284.5 eV corresponding to the graphite composition, and a weak peak at 290.5 eV which is attributed to the π-π* transition of carbon in the aromatic ring [26]. The C1s spectrum of uCNT was deconvoluted into four peaks at 288.7 eV (O=C–OH peak), 286.5 eV (C=O peak), 285.5 eV (C–OH peak), and 288.7 eV (C-C peak) [27]. The carbonyl groups were formed by oxidation attacking the bond between carbon atoms on the surface of multi-walled CNTs, resulting in the unzipping of CNTs. That is the reason the C-C peak became weak and the π-π* peak disappeared for uCNTs.

**Figure 7:**
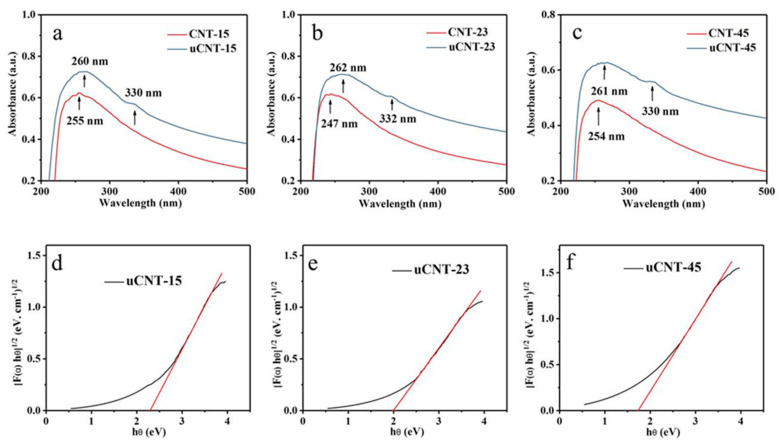
UV-Vis spectra of CNTs and uCNTs (**a**–**c**); band gap of uCNTs (**d**–**f**).

Figure 7 shows the UV-Vis spectra of CNTs and uCNTs in an aqueous solution. The UV data show that the maximum absorbance of CNTs and uCNTs was around 255 nm and 260 nm. This maximum is associated with π-π* transition for the C=C bond. For uCNTs, there was also a peak around 330 nm, which corresponds to n-π* for the C=O bond [28]. A comparison of UV-VIS spectra for CNTS and uCNTs gave further confirmation of introducing the oxygen-containing functional groups in the uCNTs obtained by oxidation with permanganate.

The band gap value of the sample was calculated by the following formula (1) for calculating the optical band gap of semiconductor materials [29]:

(1) $$\;{\left[F\left(\alpha \right)\;h\nu \right]}^{\frac{1}{2}}=\beta \;\left(h\theta -Eg\right)$$

where hν is the photon energy; *β* is a proportional constant; *Eg* is the value of the band gap; and *F*(*α*) is a Kubelka–Munk Function (2) defined as [30]

(2) $$F\left(\alpha \right)=\frac{{\left(1-R\right)}^{2}}{2R}=\frac{K}{S}$$

where *R* is the reflectivity, *K* is the absorption coefficient, and *S* is the scattering coefficient. The band gap of untreated CNTs was almost 0 eV [31]. It can be seen from the Figure 7d–f that the band gaps of uCNT-15, uCNT-23, and uCNT-45 were 2.3 eV, 2 eV, and 1.8 eV, respectively. Our study shows that changing the diameter of CNTs led to different oxidation degrees, which can change the band gap of uCNTs in a wide range of 1–2.3 eV, which is consistent with the band gap range previously proved by other researchers [28]. Moreover, increasing the diameter of pristine CNTs resulted in a decrease in the value of band gap for uCNTs.

**Figure 8:**
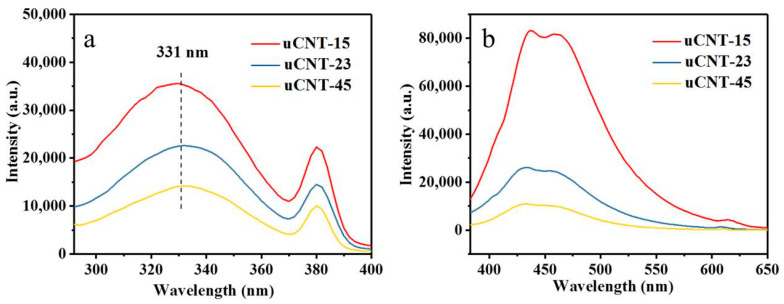
Excitation (**a**) and emission (**b**) spectra of uCNTs.

The photoluminescence spectra of uCNTs were obtained at the excitation wavelength of 331 nm at room temperature, and the emission wavelength was 436 nm. Figure 8 shows the excitation and emission spectra of uCNTs in aqueous solutions of the same concentration (0.02 mg/mL). It is worth noting that the emission spectra of the CNTs dispersions in water could not be detected in the visible light region. In contrast, the uCNTs showed strong emission in the visible light region with the main peak at 436 nm. This may be associated with electron transitions which involve molecular orbitals associated with the unsaturated carbon atoms [25,32]. The fluorescence intensity of uCNT-15 was significantly higher compared to uCNT-23 and uCNT-45. uCNTs showed an emission peak at 467 nm, which may be associated with the oxygen-containing functional groups [33]. The fluorescence at 610 nm is related to the energy of the excitation light captured by uCNTs at their defects [6]. These defects appeared because the structure of pristine CNTs was destroyed during unzipping. With the decrease of the CNTs’ diameter, the number of oxygen-containing groups and defects in uCNTs increased and the band gap increased, which led to the enhancement of fluorescence intensity. Therefore, the strongest increase of fluorescence in the unzipped CNT may be associated with the presence of oxygen-containing functional groups and defects. For CNT-15, the structure was destroyed most thoroughly, the number of oxygen-containing groups was the largest, and the fluorescence intensity was the highest.

**Figure 9:**
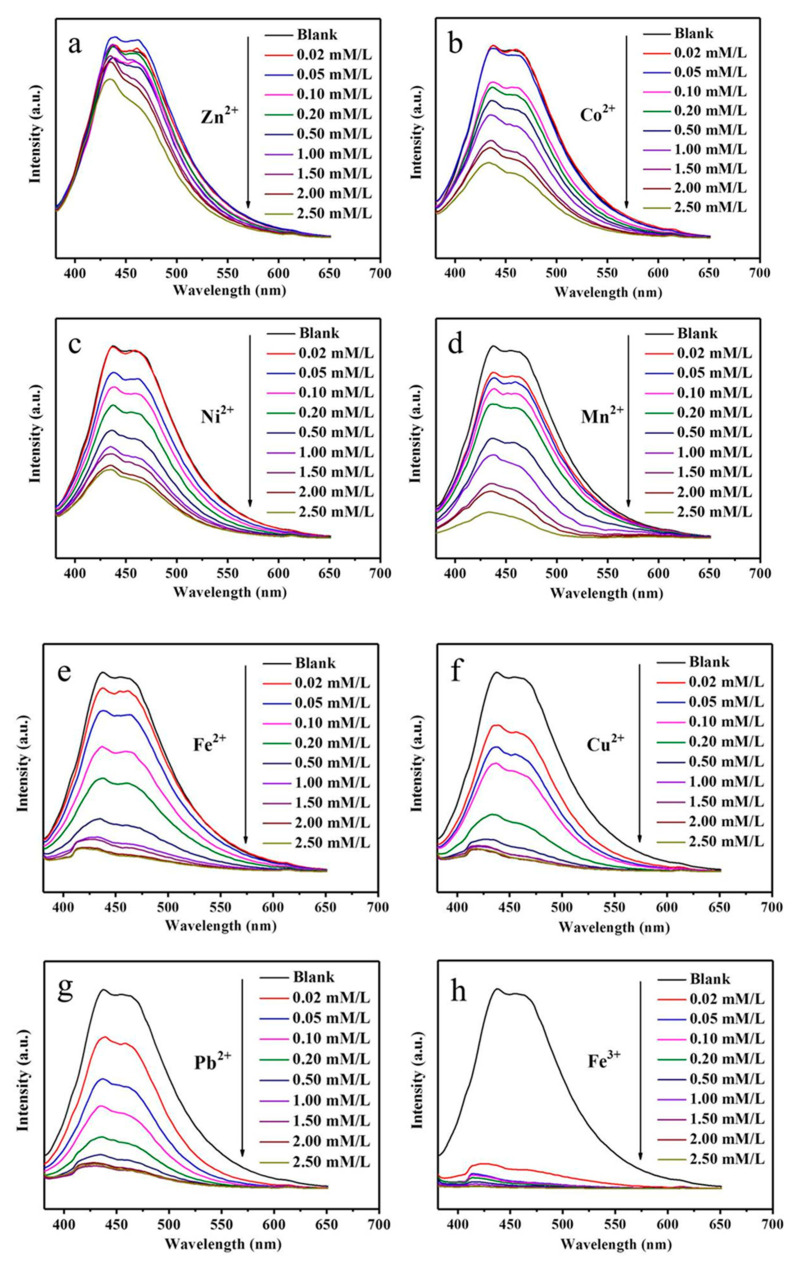

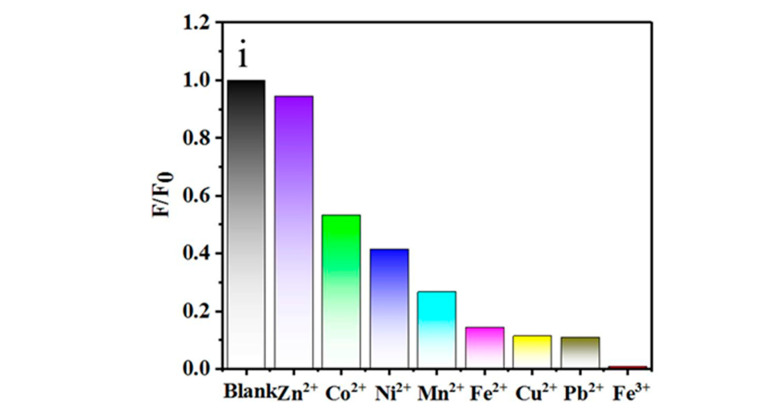
PL emission spectra of uCNT-15 in the presence of Zn^2+^ (**a**), Co^2+^ (**b**), Ni^2+^ (**c**), Mn^2+^ (**d**), Fe^2+^ (**e**), Cu^2+^ (**f**), Pb^2+^ (**g**), and Fe^3+^ (**h**) at different concentrations (mM/L). (**i**): Relative fluorescence intensity of uCNT-15 in the presence of different metal ions (F/F_0_, where F and F_0_ represent the emission intensity of uCNT-15 in the presence and absence of the test substance, respectively). The concentration of all tested species was 1.5 mM/L.

Metal ions are often used for fluorescence quenching. Particularly, metal ions can promote the selective aggregation of large aromatic molecules, which in turn leads to fluorescence quenching [34]. To show the effect of metal ions on the fluorescence intensity of uCNTs, we selected uCNT-15, which exhibited the highest photoluminescence. Figure 9 presents the photoluminescence emission spectra of uCNT-15 in the presence of different concentrations of Zn^2+^, Co^2+^, Ni^2+^, Mn^2+^, Fe^2+^, Cu^2+^, Pb^2+^, and Fe^3+^. Increasing the metal ion concentration resulted in a decrease in fluorescence intensity for all studied systems. The effect strongly depended on the nature of metal ion as follows from the data presented in Figure 9i. Zinc and cobalt showed a low quenching effect, whereas Fe^3+^ exhibited the highest quenching effect. Taking into account that the metal ions used to study the fluorescence quenching give the most common impurities, our results indicate that the fluorescence of uCNTs is not associated with impurities. Moreover, the effect of metal ions on the photoluminescence of uCNTs verifies that uCNTs exhibit self-photoluminescence.

## 4. Conclusions

The unzipping of CNTs has been discussed in the literature as a promising tool to obtain new forms of nanocarbon with unique and specific properties. Our study gives further progress in this direction showing that uCNTs exhibit self-photoluminescence. This has been illustrated for the unzipping of MWCNTs of different diameters according to the modified Hummers method. Characterization of uCNTs by XRD, XPS, FTIR, and Raman spectroscopy revealed the effect of diameter of pristine CNTs on the level of edge etching, the morphology of uCNTs, and the introduction of oxygen-containing functional groups. The band gap of uCNTs showed that oxygen-containing functional groups can open the energy level of CNTs, increasing the band gap which depends on the diameter of pristine CNTs. As the diameter of CNTs decreased, the effect of edge etching increased, which also led to an increase in the oxygen content. The photoluminescence study showed that uCNTs exhibit a good emission in the visible light region. The uCNT with the largest band gap and the highest oxygen content had the largest fluorescence intensity. Moreover, different metal ions showed different degrees of fluorescence quenching for uCNT-15, which verified the self-photoluminescence of uCNT. As a result, our study highlights that the unzipping of CNT gives an appropriate approach to obtain carbon nanostructures with tunable optical and photoluminescent properties.

## Figures and Tables

**Table 1:** The values of average diameters CNTs, the average width of uCNTs, and etching level.

Sample	D, Diameter of CNT, nm	FWHM for D, nm	W, Width of uCNT, nm	FWHM for W, nm	E = (1 − W/πD) × 100,%
CNT-15	14.88 ± 0.11	3.7	28.12 ± 0.57	20.3	40 ± 1
CNT-23	22.84 ± 0.76	10.5	34.90 ± 0.57	20.2	51 ± 3
CNT-45	45.04 ± 0.17	5.8	62.22 ± 0.75	32.5	56 ± 1

**Table 2:** Full width at half maximum of CNTs and uCNTs.

Sample	2θ (deg)	FWHM (deg)
CNT-15	26.1	1.379
	43.0	2.0
uCNT-15	25.8	7.2
	43.26	2.3
CNT-23	26.02	1.039
	43.01	1.15
uCNT-23	25.9	6.15
	42.92	2.0
CNT-45	26.0	0.997
	43.0	1.1
uCNT-45	25.78	5.5
	42.92	1.8

**Table 3:** Element percentage distributions of CNT-15, uCNT-15, CNT-23, uCNT-23, CNT-45, and uCNT-45.

Sample	C (%)	O (%)	O/C
CNT-15	99.54	0.46	0.0046
uCNT-15	80.82	19.18	23.73
CNT-23	99.56	0.44	0.0044
uCNT-23	83.04	16.96	20.42
CNT-45	99.59	0.41	0.0041
uCNT-45	86.3	13.7	15.87

## Data Availability

Not applicable.
